# Beyond the Surface: Revealing the Concealed Effects of Hyperglycemia on Ocular Surface Homeostasis and Dry Eye Disease

**DOI:** 10.3390/medicina61111992

**Published:** 2025-11-06

**Authors:** Marco Zeppieri, Matteo Capobianco, Federico Visalli, Mutali Musa, Alessandro Avitabile, Rosa Giglio, Daniele Tognetto, Caterina Gagliano, Fabiana D’Esposito, Francesco Cappellani

**Affiliations:** 1 Department of Ophthalmology, University Hospital of Udine, 33100 Udine, Italy; 2 Department of Medicine, Surgery and Health Sciences, University of Trieste, 34129 Trieste, Italy; 3 Department of Ophthalmology, University of Catania, 95123 Catania, Italy; 4 Department of Optometry, University of Benin, Benin 300283, Nigeria; 5 Department of Ophthalmology, Africa Eye Laser Center Ltd., Benin 300211, Nigeria; 6 Faculty of Medicine, University of Catania, 95123 Catania, Italy; 7 Department of Medicine and Surgery, University of Enna “Kore”, 94100 Enna, Italyfrancesco.cappellani@unikore.it (F.C.); 8 Eye Center “G.B. Morgagni-DSV”, 95125 Catania, Italy; 9 Imperial College Ophthalmic Research Group [ICORG] Unit, Imperial College, London NW1 5QH, UK

**Keywords:** diabetes mellitus, dry eye disease, hyperglycemia, corneal nerves, ocular surface

## Abstract

*Background and Objectives*: Dry eye disease (DED) is a multifactorial ocular surface disease that markedly diminishes quality of life. Although diabetes mellitus is well-known for its retinal consequences, anterior segment symptoms including dry eye disease are often overlooked. Chronic hyperglycemia causes metabolic, neurovascular, and immunological changes that undermine tear film stability, corneal innervation, and ocular surface integrity. This review seeks to consolidate existing knowledge regarding the concealed impacts of diabetes on ocular surface homeostasis, highlighting processes, diagnostic difficulties, and treatment prospects. *Materials and Methods*: A narrative review of the literature was performed by searching PubMed for publications from January 2020 to July 2025 using the terms “diabetic dry eye,” “hyperglycemia AND ocular surface,” “tear proteomics AND diabetes,” “corneal nerves AND diabetes,” and “neurotrophic keratitis.” Eligible studies were experimental research, clinical trials, and translational investigations concerning tear film function, corneal neuropathy, inflammatory indicators, or lacrimal gland dysfunction in diabetes. The exclusion criteria were non-English language, lack of primary data, and inadequate methodological description. *Results*: Hyperglycemia compromises lacrimal gland functionality, modifies lipid secretion from Meibomian glands, and diminishes corneal nerve density, resulting in neurotrophic deficits. Inflammatory cytokines and oxidative stress compromise epithelial integrity, but proteome alterations in tears serve as sensitive indicators of disease. Diagnosis is impeded by corneal hypoesthesia, resulting in a disconnection between symptoms and findings. Progress in imaging, proteomics, and artificial intelligence may facilitate earlier detection and improved risk assessment. Novel therapeutics, such as neurotrophic drugs, antioxidants, and customized anti-inflammatory approaches, show promise but remain under clinical evaluation. *Conclusions*: Diabetes-related dry eye disease is a multifaceted and underappreciated condition influenced by systemic metabolic dysfunction. The ocular surface may act as an initial indicator for systemic disease load. Narrative synthesis emphasizes the necessity for customized diagnostic instruments, individualized treatment approaches, and collaborative management. Reconceptualizing diabetic dry eye disease within the context of systemic metabolic care presents prospects for precision medicine strategies that enhance both ocular and systemic results.

## 1. Introduction

Dry eye disease (DED) is conventionally defined as a multifactorial disease of the ocular surface, characterized by instability of the tear film, pain, and visual impairment. DED represents a significant global health issue, affecting an estimated 5–30% of the population worldwide, with prevalence rising with age and comorbidities such as diabetes. Beyond its ocular implications, DED imposes a considerable socioeconomic burden due to its chronic nature and its impact on visual function and quality of life. DED can arise in the general population through a multifactorial interplay of age-related changes, hormonal dysregulation, environmental stress, and immune-mediated mechanisms. These factors contribute to lacrimal gland hypofunction, meibomian gland obstruction, and epithelial instability, leading to tear film hyperosmolarity and chronic low-grade inflammation. While typically linked to autoimmune diseases, aging, or environmental factors, its occurrence in metabolic disorders like diabetes mellitus is becoming increasingly recognized [1]. Diabetes, a chronic condition impacting over 500 million individuals globally, induces a diverse range of ocular problems. Although diabetic retinopathy and cataract are the principal concerns of clinical focus, anterior segment problems including DED are frequently overlooked [2].

Increasing recognition indicates that hyperglycemia triggers several metabolic and neuroinflammatory alterations that extend beyond the retina, profoundly impacting ocular surface homeostasis. Diabetic individuals frequently exhibit subclinical or atypical signs of dry eye disease, potentially delaying diagnosis and hindering therapy efficacy. This delay is frequently exacerbated by diminished corneal sensitivity, which mitigates clinical symptoms despite persistent epithelial and neuronal damage [3]. Studies in patients with diabetes report variable prevalence of DED depending on setting, diagnostic criteria and diabetes-control status. For example, one large meta-analysis including approximatively 2.5 million individuals found that diabetes was associated with 30% increased odds of DED (OR 1.30; 95% CI 1.08–1.57) [4]. In a hospital-based Indian cohort of 210 eyes from type 2 diabetics, the DED prevalence was 43.8%, with a strong link to poor glycemic control, longer disease duration and proliferative retinopathy [5]

By contrast, a community-based Chinese study found a lower prevalence of 17.5% in type 2 diabetic patients [6]. These differences presumably reflect setting (hospital vs. community), diagnostic cut-offs, metabolic control and duration of diabetes. Consequently, diabetic eye disease is both inadequately recognized and undervalued in diabetic communities. Significantly, changes in the ocular surface may occur prior to posterior segment disease, presenting an opportunity for early intervention. By concentrating clinical attention on the anterior segment, doctors may identify systemic metabolic abnormalities via ocular assessment. The ocular surface functions as an easily accessible and observable biomarker of systemic disease load. Moreover, advanced technologies and proteomic methodologies have facilitated a more intricate examination of tear film composition, uncovering numerous biomarkers associated with metabolic regulation and inflammation [7,8].

This review seeks to examine the hidden yet substantial effects of hyperglycemia on the ocular surface. It will incorporate recent findings from experimental and clinical research to highlight the mechanisms underlying lacrimal dysfunction, alterations in tear proteomics and corneal neuropathy. The study advocates for a prospective outlook on diagnostic innovation and therapeutic strategies, emphasizing the necessity for tailored care in diabetic patients with DED.

## 2. Materials and Methods

This evaluative review is founded on a comprehensive synthesis of both experimental and clinical research. The primary materials were obtained from PubMed focused on the timeframe from January 2020 to July 2025. Search terms comprised “diabetic dry eye,” “hyperglycemia AND ocular surface,” “tear proteomics AND diabetes,” “corneal nerves AND diabetes,” and “neurotrophic keratitis.” The literature evaluation encompassed original research publications, clinical trials, reviews, and translational studies pertinent to the etiology and therapeutic management of diabetic ocular surface disease.

Studies were chosen according to these inclusion criteria: publication in peer-reviewed journals indexed in PubMed; relevance to ocular surface alterations associated with diabetes mellitus; and the availability of data concerning tear film functionality, corneal nerves integrity, inflammatory markers, or lacrimal gland pathology. Articles not available in English, those without primary data, or exhibiting unclear methodological reporting were removed. Significant attention was placed on research involving human cohorts, diabetic animal models, and sophisticated imaging or proteomic investigations.

A total of 172 articles were initially obtained from PubMed between January 2020 and July 2025 utilizing the designated search terms. Following the elimination of 36 duplicates, 136 titles and abstracts were evaluated for eligibility. Seventy-three articles were excluded for failing to meet the inclusion criteria, which included non-English language, lack of primary data, or inadequate methodological detail. Ultimately, 63 studies met all requirements and were incorporated into the current narrative synthesis. The selection and screening were conducted independently by two reviewers (M.Z. and F.V.), with any discrepancies addressed through discussion with a third reviewer (C.G.). Considerable focus was directed towards studies involving human cohorts, diabetic animal models, and advanced imaging or proteomic analyses.

The methodological quality of the included studies was assessed qualitatively instead of utilizing a formal bias evaluation instrument. Each study was assessed for its relevance to the review objectives, clarity of design, completeness of data reporting, and alignment of outcomes with established diagnostic or experimental standards. Priority was assigned to peer-reviewed studies that provided original data, explicit methods, and specified endpoints. Review articles were included solely to furnish contextual background. This procedure guaranteed the inclusion of only those studies characterized by sufficient methodological transparency and scientific trustworthiness in the final synthesis.

## 3. Discussion

### 3.1. Historical Overview of Diabetic Dry Eye

**Figure 1:**
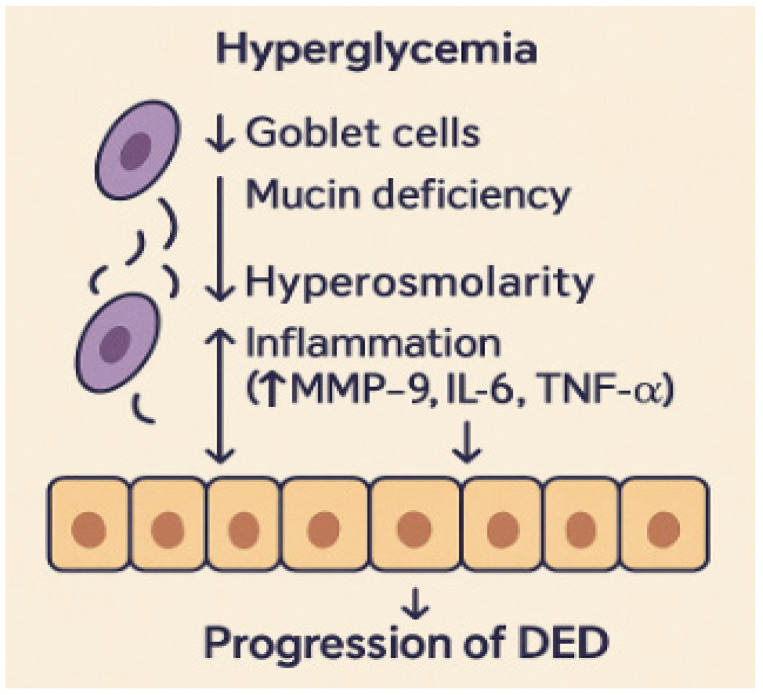
Schematic representation of how goblet cell loss, mucin insufficiency, and impaired epithelial turnover contribute to the pathogenesis of dry eye disease.

The association between diabetes mellitus and ocular surface disease has traditionally been insufficiently investigated, with the majority of clinical attention concentrated on diabetic retinopathy and cataracts. Initial clinical findings indicated that diabetic patients commonly had nonspecific eye discomfort, including burning, tears, and variable vision, which were frequently regarded as secondary symptoms [9]. In the early 2000s, a systematic approach emerged, revealing reduced tear production in diabetes patients relative to non-diabetic controls, indicating an inherent link between hyperglycemia and tear film malfunction [10]. Multiple studies have demonstrated that loss of goblet cells, mucin insufficiency, and slowed epithelial regeneration are key contributors to ocular surface dysfunction in diabetes [11]. These alterations critically affect tear film homeostasis and ocular surface protection. Goblet cells are the main source of mucins, which form the innermost tear layer, ensuring tear film stability and epithelial hydration. A reduction in goblet cell density and mucin secretion leads to an unstable and hyperosmolar tear film, increasing friction and mechanical stress on the corneal epithelium. At the same time, impaired epithelial turnover limits regenerative capacity and compromises the corneal barrier, facilitating inflammatory cytokine infiltration and accelerating surface damage. Together, these processes establish a cycle of instability, inflammation, and epithelial stress that underlies the chronic progression of diabetic dry eye disease [12]. Figure 1 illustrates how goblet cell loss, mucin deficiency, and impaired epithelial turnover under hyperglycemic conditions contribute to inflammation and the progression of dry eye disease.

Advancements in molecular understanding have revealed how metabolic dysregulation in diabetes affects lacrimal gland function, corneal epithelial integrity, and immunological homeostasis. Shetty et al. indicated that hyperglycemia adversely affects lacrimal gland acinar structure and diminishes peroxidase secretion, thereby demonstrating a biochemical connection between systemic disease and localized tear dysfunction [13]. Additional research indicated that oxidative stress mechanisms, including as mitochondrial failure and the buildup of advanced glycation end-products, compromise the ocular surface barrier [14,15]. The findings indicated that diabetes dry eye is complex, resulting from the interplay of oxidative stress, inflammation, and neurovascular impairment. These findings established the foundation for acknowledging diabetes dry eye as a systemic-ocular interface condition.

Technological developments, such as in vivo confocal microscopy (IVCM), have further advanced the field by facilitating the detection of corneal nerve changes in diabetic patients. Lagali et al. revealed that diminished nerve fiber density and branching in the sub-basal plexus were prevalent in type 2 diabetes and associated with disease duration [16]. The findings linked neuropathy to ocular surface disease by demonstrating that compromised corneal innervation diminished reflex tearing and impeded epithelial wound repair. Significantly, these imaging technologies offered quantifiable measurements, making diabetic ocular surface disease objectively quantifiable.

By the mid-2010s, narrative and systematic studies consolidated decades of data, confirming diabetes dry eye as a clinically separate condition. Misra and associates examined evidence connecting diabetes to tear malfunction, corneal neuropathy, and ocular surface inflammation, underscoring the necessity for comprehensive diagnostic methodologies [17]. Their findings emphasized that diabetic dry eye should no longer be seen as a trivial side effect, but rather as a significant component of diabetic eye disease, affecting visual function and quality of life. However, early studies were limited by small sample sizes, heterogeneous diagnostic definitions, and lack of standardized dry eye criteria, which constrained comparability and generalizability. The historical progression of research indicates a transition from disregard to acknowledgment, culminating in the current phase of translational and mechanistic investigation.

### 3.2. Epidemiology and Worldwide Impact

DED is among the most common ocular disorders globally, with population-based studies indicating prevalence rates between 5% and 34%, influenced by geographic location, age, and diagnostic criteria. This variability largely arises from methodological differences, as some studies rely on symptom-based questionnaires such as the Ocular Surface Disease Index (OSDI), whereas others use objective tear function parameters such as Schirmer or tear breakup time [18]. Diabetes mellitus markedly elevates the risk of dry eye disease, since numerous cross-sectional and cohort studies have demonstrated that both type 1 and type 2 diabetic individuals exhibit increased rates of ocular surface dysfunction. Kuo et al. conducted a meta-analysis indicating that tear breakup time and Schirmer test scores were significantly lower in diabetic populations than in controls, with these disparities being more pronounced in cases of poorly managed diabetes [19]. These data indicate that metabolic state directly affects ocular surface outcomes.

The worldwide epidemiological impact of diabetic dry eye illustrates the increasing prevalence of diabetes and the inconsistency in clinical identification. In areas with elevated diabetes prevalence, such as South Asia and the Middle East, studies indicate notably high occurrences of dry eye symptoms among diabetic individuals [20]. Environmental factors, including air pollution, nutritional condition, and healthcare access, influence prevalence estimates, highlighting the multidimensional character of disease burden [21]. Furthermore, diabetes dry eye frequently manifests before diabetic retinopathy, offering a distinct opportunity for early diagnosis and care.

Epidemiological studies reveal both structural and functional links between dry eye and systemic diseases, in addition to prevalence. Additional studies demonstrated that patients exhibiting poor metabolic control, as evidenced by raised HbA1c levels, experienced increased meibomian gland dropout and lacrimal gland dysfunction; however, this analysis was simple correlation only and did not adjust for key confounders such as age, sex or diabetes duration [22]. These findings underscore the ocular surface as a critical indicator of systemic metabolic dysregulation.

The worldwide impact of diabetic dry eye encompasses not just symptoms but also economic and quality-of-life implications. Patients with concurrent diabetes and dry eye disease experience more significant challenges in daily activities, including reading, driving, and using digital screens, compared to non-diabetic DED patients [23]. Healthcare utilization for dry eye is markedly elevated in diabetes individuals, indicating the chronic nature and resistance of the disease. Survey-based cost analyses have shown that the mean annual cost for managing DED is approximately USD $783 per patient, underscoring the substantial healthcare burden associated with the disease [24]. These findings collectively demonstrate diabetes dry eye as a significant public health and economic issue, underscoring the necessity for coordinated screening and care techniques.

### 3.3. Hyperglycemia-Induced Dysfunction of Tear Film and Lacrimal Glands

The lacrimal gland is essential for preserving tear homeostasis and the integrity of the ocular surface. In diabetes mellitus, persistent hyperglycemia significantly impairs the structure and function of the lacrimal gland. Research on animals has demonstrated that diabetic rodents display acinar cell atrophy, periductal fibrosis, and reduced glandular secretory function [8]. These modifications impair tear production and disturb the biological nature of the tear film. Recent studies indicate that the impairment of lacrimal gland function in diabetes may be partially due to oxidative stress and mitochondrial malfunction. A recent work using leptin receptor-deficient diabetic mice revealed modified circadian expression of clock genes in lacrimal tissue, establishing a connection between metabolic dysregulation and disruptions in secretory rhythms [25]. This discovery highlights a new neuroendocrine mechanism via which hyperglycemia disrupts lacrimal function, directly affecting tear film volume and composition.

Diabetic patients exhibit markedly reduced Schirmer test scores and elevated tear osmolarity relative to age-matched non-diabetic controls [26,27]. These abnormalities frequently arise independently of diabetic retinopathy, indicating that tear dysfunction is an early and independent expression of metabolic imbalance. Additionally, inflammatory cytokines including IL-6 and TNF-α have been identified in the lacrimal gland tissue of diabetic animals, suggesting local immune activation in the development of tear insufficiency [28]. However, since systemic low-grade inflammation is also a hallmark of diabetes, it remains uncertain to what extent the observed cytokine elevation reflects localized lacrimal gland pathology versus systemic inflammatory spillover. The concurrent presence of both mechanisms likely contributes to the chronic ocular surface inflammation observed in diabetic dry eye.

Alongside diminished aqueous production, diabetes individuals often have evaporative dry eye resulting from Meibomian gland dysfunction. Hyperglycemia-induced alterations in lipid production and glandular structure compromise the lipid layer of the tear film, worsening tear instability [29,30]. Imaging investigations employing non-contact meibography have demonstrated gland dropout and atrophy in diabetic individuals, associated with disease duration and inadequate metabolic management [31,32]. Novel treatment approaches aimed at preserving lacrimal function are currently being explored. This encompasses topical antioxidants, insulin-mimetic peptides, and regenerative biologics designed to restore glandular structure and function [33,34,35]. Clinical trials evaluating the effectiveness of these therapies remain scarce, highlighting the necessity for translational research that connects experimental findings to clinical practice.

### 3.4. Corneal Neuropathy and Neurotrophic Deficiencies in Diabetic Dry Eye

The cornea is one of the most densely innervated tissues in the human body and is essential for preserving ocular surface health through its sensory, trophic, and defensive activities. In patients with diabetes, peripheral neuropathy is among the earliest and most prevalent sequelae of hyperglycemia, with the cornea being especially vulnerable. IVCM has revealed structural alterations in the sub-basal nerve plexus, characterized by significant decreases in nerve fiber length, density, and branching. Lagali et al. quantified a mean corneal nerve fiber density of 13.1 mm/mm^2^ in patients with type 2 diabetes versus 15.0 mm/mm^2^ in non-diabetic controls [16]. These modifications are strongly associated with the duration of diabetes and the occurrence of further microvascular problems, including nephropathy and neuropathy. The functional repercussions of corneal nerve loss in diabetes encompass diminished corneal sensitivity (hypoesthesia), compromised blink reflex, and protracted epithelial wound healing. These alterations combined facilitate the emergence of a particular subtype of dry eye disease referred to as neurotrophic dry eye. Patients with this disease frequently display little symptoms despite considerable epithelium damage, complicating diagnosis and heightening the risk of additional infections or enduring epithelial abnormalities [36].

Recent studies indicate that hyperglycemia disrupts axonal transport and Schwann cell functionality, resulting in demyelination and degeneration of ocular nerves. Oxidative stress and the buildup of advanced glycation end products (AGEs) are crucial factors in the neurodegenerative process [37]. These pathways have been replicated in streptozotocin-induced diabetic mouse models, which exhibit analogous structural and functional impairments, rendering them significant platforms for therapeutic investigation [38]. Initiatives to restore corneal nerve integrity have concentrated on the utilization of neurotrophic drugs, including nerve growth factor (NGF), ciliary neurotrophic factor (CNTF), and insulin-like growth factor-1 (IGF-1) [39]. Cenegermin, a recombinant form of human nerve growth factor, has demonstrated effectiveness in the treatment of neurotrophic keratitis and is currently being studied for wider uses in diabetic ocular surface disease [40]. Preliminary trials indicate that these compounds may enhance corneal sensitivity and support epithelial integrity, especially when used with lubricants and anti-inflammatory drugs. However, their high cost and limited availability may currently restrict widespread clinical use.

Corneal esthesiometry, IVCM, and quantitative sensory testing are now suggested as methods to evaluate corneal nerve function and track therapy efficacy. These objective techniques hold potential for enhancing diagnosis, stratifying risk, and customizing treatment. Corneal nerve measurements may serve as surrogate indicators for systemic diabetic neuropathy, thereby connecting ocular evaluations to comprehensive disease treatment. Incorporating these approaches into standard diabetic eye care constitutes a progressive approach to enhance patient outcomes. Given their potential to mirror broader neurodegenerative alterations, further investigation of corneal nerve metrics as potential systemic biomarkers may represent an important future research direction.

### 3.5. Inflammation, Oxidative Stress, and Tear Proteomics in Diabetic Ocular Surface Pathology

#### 3.5.1. Inflammatory Pathways and Cytokine Dysregulation

Systemic low-grade inflammation is a characteristic of type 2 diabetes [41] and directly impacts the ocular surface. Chronic hyperglycemia results in an increase in inflammatory cytokines, including interleukin-1β (IL-1β), tumor necrosis factor-alpha (TNF-α), and matrix metalloproteinase-9 (MMP-9) in tears and conjunctival tissues. These molecular changes are mechanistically linked to disruption of epithelial barrier integrity, reduction in goblet cell density, and increased immune cell infiltration [42].

#### 3.5.2. Oxidative Stress and Mitochondrial Dysfunction

**Figure 2:**
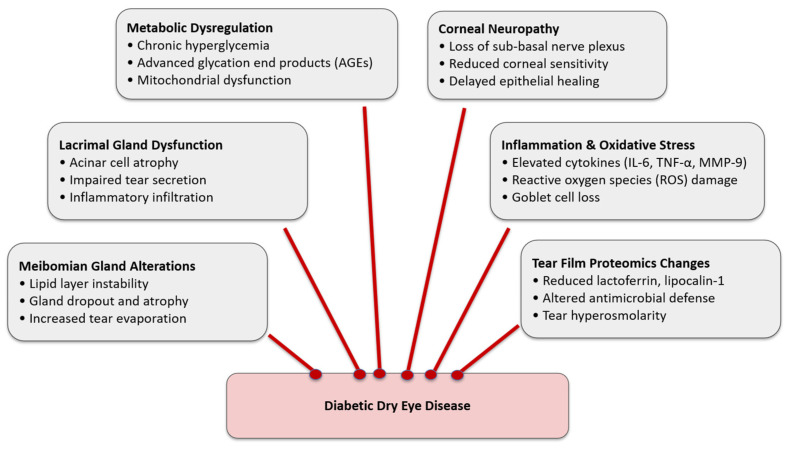
Pathophysiological mechanisms behind diabetic dry eye disease. Schematic depiction of the multiple routes via which diabetes mellitus influences ocular surface homeostasis.

Oxidative stress collaborates with inflammation in the development of diabetes dry eye. Clinically, these processes are associated with ocular surface instability and symptomatic dry eye in diabetic patients [42]. Hyperglycemia promotes oxidative stress and inflammatory cascades within ocular tissues through multiple mechanisms, including activation of the polyol and hexosamine pathways, accumulation of AGEs, and stimulation of protein kinase C. These processes increase reactive oxygen species generation and proinflammatory cytokine release, leading to endothelial dysfunction, microvascular compromise, and tissue injury [43]. The interconnected mechanisms underlying lacrimal dysfunction, corneal neuropathy, meibomian gland alterations, inflammation, and tear proteomic changes in diabetes are summarized schematically in Figure 2.

Mitochondrial failure in ocular surface epithelial cells results in excessive generation of reactive oxygen species (ROS), causing damage to cellular proteins, lipids, and DNA. In diabetic animal models and human cells, increased reactive oxygen species levels in the corneal and conjunctival epithelia have been linked to epithelial thinning, impaired wound healing, and compromised tight junction integrity [44]. Antioxidant defense mechanisms, such as superoxide dismutase and glutathione peroxidase, frequently prove inadequate in mitigating oxidative stress in poorly managed diabetes [14].

#### 3.5.3. Tear Proteomics and Biomolecular Insights

Recent advancements in tear proteomics have elucidated the molecular foundations of diabetic ocular surface pathology. Mass spectrometry analysis have identified alteredexpression of proteins including lactoferrin, lipocalin-1, and lysozyme, which are crucial for tear stability and antimicrobial defense. Although tear proteomics provides promising diagnostic insights, its clinical translation remains constrained by variability in sample collection, analytical reproducibility, and limited accessibility of mass spectrometry platforms. Standardization of protocols and validation across centers are essential for future clinical adoption [8,45]. Summary of studies on tear proteomics and corneal nerve alterations in diabetic ocular surface disease is provided in Table 1.

Clinical studies have revealed substantial disparities in tear film composition between diabetic and non-diabetic people. Tear osmolarity and MMP-9 levels are significantly increased in diabetic individuals exhibiting dry eye symptoms, even without severe retinopathy [47,48]. The findings indicate that the tear film may function as a sensitive and non-invasive indicator for early ocular surface involvement in systemic metabolic disease. Integrating tear analysis with conventional diabetic eye evaluations may thus enable earlier identification and intervention. Therapeutically, managing inflammation and oxidative stress on the diabetic ocular surface presents significant challenges. Antioxidant treatments, including vitamin C, omega-3 fatty acids, and coenzyme Q10, have shown measurable improvements in tear film stability and ocular surface parameters in small interventional studies, although the overall benefit remains modest and requires confirmation in larger controlled trials [50]. Future therapeutics may incorporate customized interventions derived from tear proteome profiling, facilitating personalized therapy protocols that target the distinct inflammatory and oxidative characteristics of each patient. The incorporation of tear proteomics into clinical practice represents a significant transformation in the diagnosis and management of diabetes dry eye. Molecular insights provide precision diagnoses, risk stratification, and dynamic monitoring of therapy efficacy, surpassing conventional symptom-based assessments. Ongoing research in this domain is crucial for converting these findings into accessible and dependable therapeutic instruments. Beyond oxidative stress and inflammation, systemic metabolic factors also contribute to diabetic ocular surface disease. Insulin resistance and altered adipokine signaling may disrupt epithelial metabolism and tear film homeostasis, while decreased levels of neurotrophic and growth factors such as NGF and epidermal growth factor (EGF) can impair corneal nerve regeneration and epithelial healing. These mechanisms highlight the close interplay between systemic metabolic dysfunction and local ocular surface pathology in diabetes [3,51].

### 3.6. Diagnostic Difficulties and Imaging Advancements

Diagnosing dry eye disease in diabetes individuals is hampered by the frequent discordance between clinical manifestations and subjective symptoms. Corneal hypoesthesia resulting from neuropathy leads many patients to neglect reporting discomfort or grittiness, even when there is considerable ocular surface damage [9]. Conventional surveys, including the OSDI, may thus undervalue the disease burden in this demographic. Clinicians must increasingly depend on objective diagnostic instruments and uphold a heightened level of suspicion in silent diabetes patients. Traditional diagnostic assessments, such the Schirmer test and tear breakup time (TBUT), offer limited information regarding tear volume and stability but exhibit limited sensitivity for early detection or neurotrophic variants of dry eye. Fluorescein, rose bengal, or lissamine green staining techniques can identify epithelial abnormalities, although they may fail to detect modest pathological alterations. Consequently, novel diagnostic techniques are required to enhance the identification and surveillance of diabetic ocular surface disease.

IVCM has become an essential instrument for assessing corneal nerves, epithelial cell density, and immune cell infiltration at a microscopic scale. Multiple investigations have evidenced the efficacy of IVCM in detecting early nerve fiber degeneration, heightened dendritic cell density, and epithelial abnormalities in diabetic eyes. These alterations frequently occur before to clinical manifestations and correlate with inadequate glycemic regulation and disease duration, underscoring the significance of IVCM as a biomarker for subclinical pathology [52,53,54]. However, interpretation of corneal nerve parameters derived from IVCM should consider methodological variability across studies. Differences in imaging protocols, magnification, depth selection, and quantification algorithms (manual versus automated) can influence reported nerve density and morphology, limiting direct comparability. Standardization efforts are essential for reliable cross-study interpretation and clinical translation. Non-invasive imaging modalities, including anterior segment optical coherence tomography (AS-OCT), have been modified to assess tear meniscus height, corneal epithelial thickness, and conjunctival alterations. AS-OCT delivers high-resolution cross-sectional pictures for quantitative evaluation of tear volume and corneal epithelium remodeling in response to treatment [55]. When used alongside interferometry for lipid layer assessment and thermography for evaporation rate measurement, these imaging modalities can provide an extensive overview of tear film dynamics in diabetic individuals [56].

The incorporation of AI and machine learning into ocular surface diagnostics is a burgeoning domain. Algorithms trained on extensive imaging datasets can identify patterns of neuron degeneration, epithelium alterations, and tear film irregularities with enhanced precision. Pilot investigations have shown the viability of AI-assisted IVCM analysis in identifying diabetic neuropathy, possibly enabling automated, swift, and consistent evaluations [57]. As these technologies advance, they may offer economical and scalable options for the screening and monitoring of diabetic dry eye disease; however, considerations regarding data privacy, algorithm transparency, and clinical validation remain essential for their safe and ethical implementation. In low-resource settings, additional challenges include limited access to high-resolution imaging equipment, inadequate digital infrastructure, and the cost of AI integration, which collectively constrain widespread adoption.

**Figure 3:**
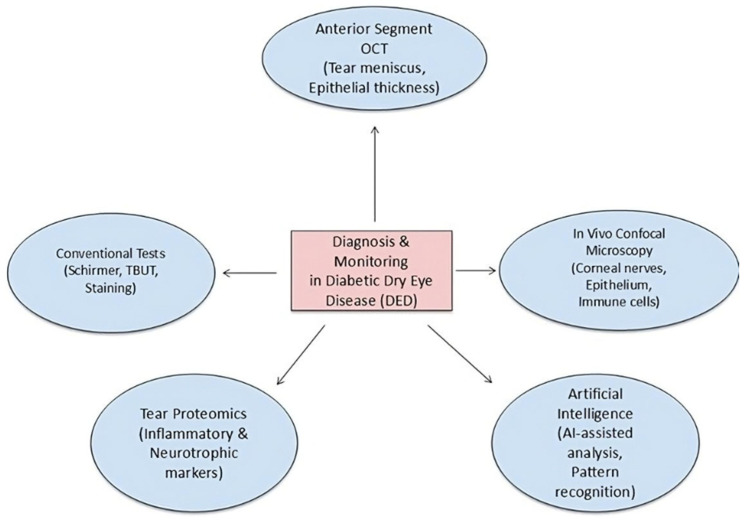
Diagnostic instruments for diabetic dry eye disease.

Nonetheless, the extensive adoption of sophisticated imaging is constrained by expenses, equipment accessibility, and the requirement for specialized training. Simplified imaging procedures and teleophthalmology platforms are being investigated to surmount these obstacles. Smartphone-compatible slit-lamp adapters, portable OCT devices, and cloud-based image analysis may facilitate equitable access to advanced diagnostics and promote early identification of ocular surface disease in remote or underserved communities [58,59]. Enhancing diagnostic precision will result in earlier intervention, superior disease management, and an elevated quality of life for individuals with diabetes. Diagnostic modalities, including in vivo confocal microscopy, anterior segment OCT, tear proteomics, and artificial intelligence–based platforms for image analysis, are illustrated in Figure 3.

### 3.7. Prospective Research Avenues: Biomarkers and Artificial Intelligence

A systematic analysis by Polkamp et al. identified increased inflammatory markers, including IL-6 and TNF-α, and reduced protective proteins such as lactoferrin, underscoring their potential diagnostic significance [60]. These biomarkers not only facilitate earlier disease identification but also offer mechanistic insights into the inflammatory and neuronal processes that underpin diabetic ocular surface disease. Furthermore, recent evidence highlights how biomarkers can enable timely detection and targeted intervention [61].

Lacritin has garnered significant interest among candidate compounds due to its multifaceted involvement in maintaining ocular surface homeostasis. Lacritin enhances tear production, epithelium viability, and corneal nerve regeneration, with its deficit associated with severe manifestations of DED [62]. Innovative topical treatments are now being investigated for diabetes dry eye. Topical insulin has been assessed in preclinical and clinical trials, demonstrating enhancements in corneal wound healing and decreases in tear film inflammatory biomarkers. Evidence from small randomized and observational studies further supports its ability to accelerate epithelial repair and partially restore corneal sensitivity. Nonetheless, current evidence is derived from limited trials, and large-scale randomized controlled studies are required to establish safety and efficacy [63]. Chen et al. shown that the topical administration of a pigment epithelium-derived factor (PEDF) peptide reinstated lacrimal gland and corneal function in diabetic mice, thereby broadening treatment possibilities; however, these findings remain limited to animal and in vitro studies and lack clinical validation [35]. Table 2 summarizes these potential emerging treatment strategies for diabetic dry eye disease, including their mechanisms of action, key findings, and principal limitations.

These methodologies advance the discipline towards mechanism-oriented therapies instead of only alleviating symptoms.

AI signifies a new frontier in the research and therapy of diabetes dry eye. AI-driven image analysis utilized in in vivo confocal microscopy and optical coherence tomography can detect tiny corneal and tear film abnormalities with more sensitivity than manual grading [56]. Preliminary research indicates that machine learning algorithms may identify patterns of diabetic neuropathy in corneal nerve pictures, implying the feasibility of automated, large-scale screening [57]. The integration of AI with tear biomarker profiles and clinical data may facilitate tailored therapy algorithms, advancing diabetes dry eye management into the realm of precision medicine, although current progress remains preliminary. There is an urgent necessity for tailored therapies that target the specific disease mechanisms in these patients. Neurotrophic drugs, antioxidant treatments, and focused anti-inflammatory strategies may exhibit significant potential. Furthermore, incorporating ocular surface evaluation into standard diabetic management can have a dual advantage—enhancing localized results while potentially acting as an early indicator of systemic control decline. Current evidence on diabetic dry eye remains limited by heterogeneous study designs, variable diagnostic criteria, and small cohort sizes, which hinder direct comparison across studies. Most available data are cross-sectional, providing limited insight into temporal progression or treatment responsiveness. Future research should prioritize longitudinal and interventional studies that integrate multimodal imaging, tear proteomics, and neurotrophic markers to better define causal mechanisms and therapeutic windows. Standardization of protocols and cost-effective diagnostic platforms will also be essential for translating emerging technologies into clinical and population-level applications.

This review is limited by the heterogeneity of the included studies, particularly in imaging protocols, biomarker assays, and diagnostic criteria for dry eye. Many cited investigations involved small sample sizes and short follow-up durations, which may restrict generalizability. As a narrative rather than systematic review, potential selection bias cannot be entirely excluded.

## 4. Conclusions

Dry eye disease in individuals with diabetes mellitus is a complicated, multifaceted, and often underappreciated syndrome that substantially impacts quality of life and visual performance. The pathophysiology involves complex interactions among chronic hyperglycemia, inflammatory mediators, neurotrophic deficiencies, and proteomic changes in the tear film, rather than merely tear shortage. These alterations indicate a more extensive systemic dysregulation, rendering the ocular surface a discernible and accessible indicator of diabetes disease burden. Impairment of the lacrimal gland, degradation of corneal nerves, activation of inflammatory cytokines, and dysregulation of tear film proteins together contribute to the distinctive clinical picture of diabetes dry eye. These processes not only aggravate ocular surface disease but also obscure early signs, postponing diagnosis and therapy. In this context, enhancing diagnostic methodologies—such as in vivo imaging, tear biomarker analysis, and AI-assisted platforms—provides a means to improve detection and management.

Existing treatment options, although beneficial in many instances, are not properly customized for the diabetic population. A transformation in therapeutic perspective is necessary. Dry eye disease in diabetes should be seen not merely as a singular ocular concern but as a systemic condition requiring interdisciplinary collaboration for integrative diabetic care. As research in this domain progresses, there exists a chance to reconfigure therapeutic paradigms and provide genuinely integrative, patient-centered ocular care.

## Figures and Tables

**Table 1:** Summary of key clinical and experimental studies examining tear proteomic and corneal nerve alterations in diabetic ocular surface disease.

Study	Sample	Analyzed Parameter(s)	Assay/Imaging Method	Main Findings
Csősz et al., 2012 [45]	119 diabetic, 26 controls	Tear proteome (lactoferrin, lipocalin-1, lysozyme, lacritin, Ig λ chain)	iTRAQ-based LC–MS/MS proteomic profiling	Identified 53 tear proteins, 15 significantly upregulated in PDR; lipocalin-1 and lactotransferrin proposed as potential biomarkers for disease progression.
Zhou et al., 2024 [8]	48 DR patients, 22 controls	Tear progranulin (PGRN), TNF-α, IL-6, MMP-9; corneal nerve morphology	Luminex multiplex assay; IVCM	Tear PGRN significantly reduced in DR; PGRN positively correlated with corneal nerve density, length, and branch density.
Lagali et al., 2017 [16]	39 diabetic, 43 controls	Corneal nerve fiber density (CNFD), length, branch density	IVCM	CNFD: 13.1 mm/mm^2^ in diabetes vs. 15.0 mm/mm^2^ in controls.
Bitirgen et al., 2014 [46]	132 diabetic eyes (no DR, NPDR, PDR) and 32 controls	CNFD, branch density, length; epithelial, stromal, and endothelial cell densities	IVCM	Corneal nerve fiber density, length, and branching were reduced even in diabetics without DR and worsened with DR severity.
Qu et al., 2025 [47]	110 diabetic patients (55 DED, 55 non-DED)	Tear MMP-9 concentration and ocular surface indices	Immunochromatographic assay	MMP-9 significantly higher in diabetic DED; correlated with symptom severity and tear instability.
Amorim et al., 2022 [48]	54 T2D patients (13 no DR, 25 NPDR, 16 PDR) and 12 controls	Tear proteome, cytokines (IL-2, IL-4, IL-5, IL-18, TNF), MMP-2/-3/-9; extracellular vesicle proteins	Multiplex immunoassay	Elevated pro-inflammatory cytokines and MMPs in DR, correlating with disease stage and highlighting oxidative and immune dysregulation.
Byambajav et al., 2023 [49]	122 subjects (T2D + DED, T2D-only, DED-only, controls)	Tear cytokines (IL-6, IL-8, IL-1RA, TNF-α, VEGF, etc.); metabolic proteins (Leptin, Insulin)	Multiplex bead immunoassay (Luminex)	IL-6 and IL-8 elevated in T2D + DED vs. all other groups; correlated with corneal staining and reduced tear stability. Suggests inflammatory cytokines as potential biomarkers of diabetic DED.

**Table 2:** Summary of emerging and approved therapeutic approaches for diabetic dry eye disease.

Therapy	Mechanism of Action	Key Findings	Limitations
PEDF Peptide (Mimetic)	Restores corneal nerve and epithelial integrity; reduces inflammatory signaling and oxidative stress	Improved corneal structure, tear secretion, and reduced inflammation in diabetic mice	Evidence limited to animal and in vitro studies
Topical Insulin	Activates insulin/IGF-1–PI3K/ERK pathways; promotes epithelial proliferation and nerve regeneration	Accelerates epithelial healing and partially restores corneal sensitivity in small clinical studies	Limited sample size and absence of large randomized trials
Cenegermin (rhNGF)	Stimulates corneal nerve regeneration and tear secretion via NGF receptor activation	Enhances corneal healing and sensitivity; approved for neurotrophic keratopathy	High cost and limited clinical accessibility

## Abbreviations

The following abbreviations are used in this manuscript:

AGEs	Advanced glycation end products
AI	Artificial intelligence
AS-OCT	Anterior segment optical coherence tomography
CNTF	Ciliary neurotrophic factor
DED	Dry eye disease
HbA1c	Glycated hemoglobin A1c
IGF-1	Insulin-like growth factor-1
IL-1β	Interleukin-1 beta
IL-6	Interleukin-6
IVCM	In vivo confocal microscopy
MMP-9	Matrix metalloproteinase-9
NGF	Nerve growth factor
OCT	Optical coherence tomography
OSDI	Ocular Surface Disease Index
PEDF	Pigment epithelium-derived factor
ROS	Reactive oxygen species
TBUT	Tear break-up time
TNF-α	Tumor necrosis factor alpha
